# Clinical Relevance of IFT140 Loss-of-Function Variants in Development of Renal Cysts

**DOI:** 10.3390/genes16050472

**Published:** 2025-04-22

**Authors:** Carlotta Pia Cristalli, Sara Calabrese, Luca Caramanna, Andrea Pietra, Giulia Vitetta, Bianca De Nicolo, Elena Bonora, Giulia Severi, Soara Menabò, Simona Ferrari, Francesca Ciurli, Valeria Aiello, Irene Capelli, Andrea Pasini, Irene Alberici, Roberto Pillon, Claudio La Scola, Cesare Rossi, Francesca Montanari, Claudio Graziano

**Affiliations:** 1 IRCCS, Azienda Ospedaliero-Universitaria di Bologna, 40138 Bologna, Italy; cesare.rossi@aosp.bo.it; 2 Department of Medical and Surgical Sciences (DIMEC), University of Bologna, 40138 Bologna, Italy; sara.calabrese7@studio.unibo.it (S.C.); luca.caramanna@studio.unibo.it (L.C.); andrea.pietra@studio.unibo.it (A.P.); giulia.vitetta@studio.unibo.it (G.V.); bianca.denicolo2@unibo.it (B.D.N.); elena.bonora6@unibo.it (E.B.); francesca.ciurli@aosp.bo.it (F.C.); irene.capelli4@unibo.it (I.C.); 3 Medical Genetics Unit, IRCCS, Azienda Ospedaliero-Universitaria di Bologna, 40138 Bologna, Italy; giulia_severi@aosp.bo.it (G.S.); soara.menabo@aosp.bo.it (S.M.); simona.ferrari@aosp.bo.it (S.F.); francesca.montanari@aosp.bo.it (F.M.); 4 Nephrology, Dialysis and Kidney Transplant Unit, IRCCS, Azienda Ospedaliero-Universitaria di Bologna, 40138 Bologna, Italy; valeria.aiello@aosp.bo.it; 5 Pediatric Nephrology and Dialysis Unit, IRCCS, Azienda Ospedaliero-Universitaria di Bologna, 40138 Bologna, Italy; andrea.pasini@aosp.bo.it (A.P.); irene.alberici@aosp.bo.it (I.A.); roberto.pillon@aosp.bo.it (R.P.); 6 Community Pediatrics Rimini and Riccione, Pediatric Nephrology, Pediatric Unit, AUSL Romagna, 47921 Rimini, Italy; 7 Medical Genetics Unit, AUSL Romagna, 47522 Cesena, Italy; claudio.graziano2@auslromagna.it

**Keywords:** *IFT140*, ESRD, ADPKD, ciliopathy

## Abstract

Background: Autosomal dominant polycystic kidney disease (ADPKD) is the most common inherited kidney disease, affecting approximately 1 in 1000 individuals. This genetically heterogeneous condition is primarily caused by monoallelic pathogenic or likely pathogenic variants in the *PKD1* and *PKD2* genes, accounting for 78% and 15% of typical cases, respectively. Recently, the application of NGS methods has led to the identification of additional genes associated with ADPKD, which have been incorporated into routine diagnostic testing for detecting phenocopies of the disease. Methods: In this study, targeted NGS (tNGS) analysis of the main cystogenes associated with classic and atypical ADPKD was performed in a cohort of 218 patients clinically diagnosed with cystic nephropathies. Results: Genetic testing identified variants in 175 out of 218 cases (80.3%). Among these, 133 probands (76%) harbored likely pathogenic or pathogenic variants in one or more genes of the panel, while 42 individuals (24%) had a variant of unknown significance (VUS). Specifically, one or more class 4/5 variants in *PKD1, PKD2*, or both were identified in 111 (83.5%) probands. Remarkably, a pathogenic variant in the *IFT140* gene was identified in 14 index cases (8% of positive individuals, 6.4% of the global cohort): 10 distinct loss-of-function (LoF) variants were identified (including four frameshift variants, four nonsense variants, and two splice site defects); one individual carried a second *IFT140* missense variant classified as VUS. Furthermore, five affected family members were found to carry a P/LP LoF variant in *IFT140*. Conclusions: Our data support that *IFT140* heterozygous *IFT140* LoF variants result in an atypical, mild form of ADPKD, consisting of bilateral kidney cysts and renal functional decline at older ages. Furthermore, we describe the second pediatric patient with a mild form of ADPKD due to an *IFT140* variant and discuss hyperuricemia as a previously unappreciated feature of this condition.

## 1. Introduction

Autosomal dominant polycystic kidney disease (ADPKD (MIM: 173900)) is the most common inherited kidney disease, affecting approximately 1 in 1000 individuals. It is characterized by the progressive formation and enlargement of renal cysts, leading to kidney enlargement and, ultimately, renal failure. ADPKD is the fourth leading cause of end-stage renal disease (ESRD) in adults worldwide [1,2,3]. ADPKD can be classified as a multisystemic disorder, as it presents with both renal and extrarenal manifestations. These include polycystic liver disease (PLD), which may occasionally require surgical intervention, and the risk of ruptured intracranial aneurysms, leading to subarachnoid hemorrhage [4,5]. ADPKD is a genetically heterogeneous condition, with monoallelic pathogenic variants in *PKD1* (encoding polycystin-1, PC1 (MIM: 601313)) and *PKD2* (encoding polycystin-2, PC2 (MIM: 173910)) accounting for approximately 78% and 15% of cases, respectively [3].

The causative gene and the type of variant (loss-of-function (LoF) vs. missense) influence disease outcomes: *PKD1*-related disease is more severe, with an average age of onset for end-stage renal disease (ESRD) at 58.0 years, compared to 74.8 years for *PKD2*-related cases. Total kidney volume (TKV), assessed via MRI, is a strong predictor of disease severity [6]. Recent advancements in next-generation sequencing (NGS) techniques, including whole-exome sequencing (WES) and targeted NGS panels (tNGS), have led to the identification of additional ADPKD-associated genes, which are now incorporated into routine diagnostic testing [7,8,9]. Recent studies have highlighted the *IFT140* gene as an important contributor to ADPKD. Senum et al. reported monoallelic pathogenic *IFT140* variants in 12 multiplex ADPKD families and 26 singleton index cases. Heterozygous loss-of-function (*LoF*) *IFT140* variants result in an atypical, mild form of ADPKD characterized by large bilateral cysts and renal functional decline at older ages [10], along with a few liver cysts. Additionally, Salhi et al. confirmed that heterozygous *IFT140* frameshift variants are responsible for a renal cystic phenotype. Their study also raised the possibility of an associated cardiac phenotype, specifically dilated cardiomyopathy. This condition was of unknown origin, as exome sequencing analysis failed to identify an alternative genetic cause, suggesting a potential link between *IFT140* and heart disease [11]. Dordoni et al. included the *IFT140* gene in a new NGS panel and retrospectively analyzed a cohort of patients with a negative ADPKD-spectrum diagnosis. Loss-of-function pathogenic variants in the *IFT140* gene were identified in three unrelated patients (2.3%) [12]. The *IFT140* gene (MIM: 614620) consists of 31 exons and has a coding region of 4386 bp (GenBank: NM_014714.4). It encodes the *IFT140* protein, composed of 1462 amino acids (GenBank: NP_055529.2). *IFT140* is a key component of the IFT-A core complex responsible for dynein-associated retrograde trafficking of proteins from the ciliary tip back to the basal cell body [13,14,15]. Bi-allelic pathogenic variants in IFT140 have been associated with the syndromic ciliopathy Short-Rib Thoracic Dysplasia 9 with or without Polydactyly (SRTD9 (MIM: 266920)), also known as Mainzer–Saldino Syndrome [16,17,18]. The SRTD9 phenotype includes retinal dystrophy, skeletal malformations (small thorax, cone-shaped epiphyses, craniofacial abnormalities, and digit malformations), and chronic kidney disease (renal cysts and fibrosis) [16,17]. Additionally, bi-allelic variants in *IFT140* are associated with non-syndromic forms of retinal dystrophies (MIM: 617781) [19].

In this study, we use a targeted NGS approach to analyze known cystogenes [20] and describe 14 Italian families exhibiting monoallelic LoF variants in *IFT140*, including the second reported case of pediatric *IFT140*-related diagnosis. Notably, 35.7% of the patients (5 out 14) also presented with hyperuricemia.

## 2. Materials and Methods

### 2.1. Patient Recruitment

A cohort of 218 patients with a clinical diagnosis of ADPKD was referred from various centers to the Medical Genetics Unit at IRCCS Azienda Ospedaliero-Universitaria of Bologna over a 15-month period (January 2023–March 2024). Genetic counseling was provided both before and after genetic testing. All patients provided informed consent for genetic analyses and the potential publication of results.

Ethical review and approval were waived for this study because sample analysis did not deviate from current clinical practice, and local policy allows reporting of clinical data provided that the patients involved have expressed their agreement through the informed consent form. Clinical and imaging data were collected through a review of medical records. Hyperuricemia was defined as a serum uric acid level > 6.8 mg/dL [21] or acute gout attack.

### 2.2. Genetic Testing

Genetic testing was performed on DNA isolated from EDTA peripheral blood using a semi-automatic Maxwell 16 instrument (Promega Corporation, Madison, WI, USA). Targeted next-generation sequencing (tNGS) was conducted using a panel of 17 polycystic kidney disease (PKD) and ciliopathy genes, as detailed in Table S1.

Raw sequencing data were transferred to the Torrent Server, where TorrentSuite™ performed alignment to a reference genome to generate FASTQ files, a Binary Alignment Map (BAM) with a corresponding Binary Alignment Index (BAI), and Variant Call Format (VCF) files. Reads were aligned to the reference genome based on Human Genome 19 (GRCh37). All VCF files were uploaded into Ion Reporter software v5.10 (Thermo Fisher Scientific Inc., Waltham, MA, USA), selecting the Annotation Variant workflow to associate each variant with its nucleotide change in the mRNA transcript, amino acid change, exon or intronic variant (IVS) location, and function.

BAM/BAI files generated after alignment were visualized using the Integrative Genome Viewer (IGV) software v2.18.2 to assess sequencing read depth, zygosity, read quality, and mapping accuracy. Variant filtering based on population frequency was performed using population databases, including ExAC, gnomAD v4.1.0 [22], 1000 Genomes [23], and dbSNP, retaining only alleles with a minor allele frequency (MAF) ≤ 0.01. Variants were then annotated according to the guidelines of the Human Genome Variation Society [24] and classified into five categories following the standards of the American College of Medical Genetics and Genomics (ACMG) [25].

To achieve this, public variant databases such as ClinVar [26] and LOVD [27], as well as online tools including VarSome Premium [28] and Franklin [29], were utilized. The potential significance of missense variants was assessed using SIFT4G, PolyPhen-2, MutationTaster, MutationAssessor, PROVEAN, FATHMM, BLOSUM, REVEL, and CADD. Large rearrangements in *PKD1* or *PKD2* genes were excluded using multiplex ligation-dependent probe amplification (SALSA MLPA kit P351 *PKD1*, Lot No. D1-0421, and P352 *PKD1-PKD2*, Lot No. E1-0421, MRC-Holland).

Exon-specific PCR primers were designed for confirmation of variants identified in *IFT140*. Primer sequences are available upon request. Segregation analysis was conducted in family members, where possible, to support the causative role of *IFT140* variants. Some patients underwent abdominal imaging via nuclear magnetic resonance imaging (MRI) and/or computed tomography (CT).

## 3. Results

From January 2023 to March 2024, 218 individuals with a clinical diagnosis of cystic nephropathy suggestive of ADPKD were evaluated (110 females and 108 males, with a mean age of 47 years at clinical diagnosis). Genetic testing identified a variant in 175 out of 218 cases (80.3%). Among these, 133 probands (76%) carried likely pathogenic or pathogenic variants in one or more genes included in the panel, while 42 cases (24%) harbored a variant of unknown significance (VUS). Specifically, in 111 (83.5%) out of 133 cases, one or more class 4/5 variants in *PKD1*, *PKD2*, or both were identified.

Within our cohort, 13 patients (9.8%) carried more than one variant, and 23 (16.5%) had causative variants in genes associated with atypical or uncommon forms of ADPKD. A molecular defect could not be detected in 43 cases (19.7%). The complete list of variants identified through tNGS is provided in Table S2. Notably, heterozygous loss-of-function (LoF) variants in *IFT140* (NM_014714.4) were identified in 14 index cases out of 133 (10.5%, 6.4% of the global cohort). These included four frameshift variants c.20_21insT (p.Gln8ProfsTer82), c.1863_1866del (p.Glu623ArgfsTer20), c.2545_2554del (p.Val849TrpfsTer24), c.2682delCinsAA (p.His894GlnfsTer58), four stop-gain variants c.919C > T (p.Arg307Ter), c.1501C > T (p.Arg501Ter), c.2500C > T (p.Arg834Ter), c.2880G > A (p.Trp960Ter), and two splice-site defects c.2399 + 1G > T and c.2766_2768 + 1del. Some variants were recurrent (Table 1).

The proband (BO-216) carrying the p.Arg307Ter variant also harbored an IFT140 missense variant classified as a VUS c.2797G > A (p.Glu933Lys). Furthermore, proband BO-199 had an additional variant in *PKD1* classified as a VUS, c.2411A > G (p.Asn804Ser), and proband BO-133 had a dual molecular diagnosis since an *HNF4A* whole gene deletion causing MODY type 1 was identified.

Table 1 summarizes the genetic coordinates of the identified variants, their frequencies in the gnomAD v4.1.0 population database, and their classification according to ACMG guidelines. Clinical features are detailed in Appendix A reported below. In summary, all probands (nine males, five females) presented with bilateral cortical renal cysts (average age at clinical diagnosis was 47 years), while liver involvement was observed in one single patient (B0-215). The detection of kidney cysts was usually accidental, e.g., during the work-up for unexplained high blood pressure, which was present in nine patients. The severity of kidney dysfunction was heterogeneous, ranging from normal kidney function to end-stage renal disease (ESRD) (Table 2).

Among the 11 patients with available data, six (54.5%) had an eGFR >60 mL/min, three (27.3%) had CKD stage IIIa, and two (18.2%) had CKD stage IV.

**Figure 1:**
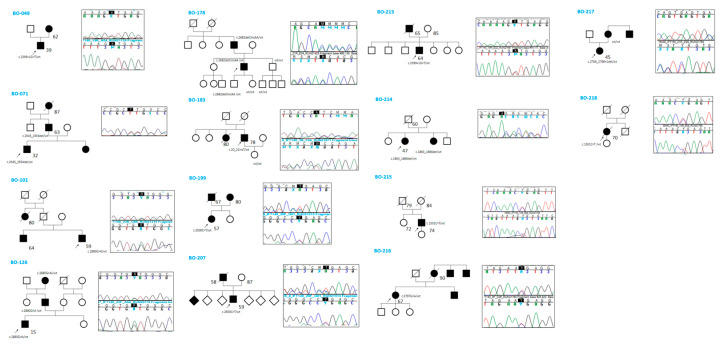
Family history and segregation analysis by Sanger sequencing.

Five cases also exhibited hyperuricemia (BO-101, BO-178, BO-207, BO-213, and BO-218)—a trait described for the first time in this study. Regarding extrarenal phenotypes, 11 out of 14 patients underwent cardiac ultrasound, and four individuals (BO-101, BO-213, BO-216, BO-217) were found to have mitral valve prolapse; however, none had dilated cardiomyopathy. Two individuals (BO-214 and BO-216) were found to have cerebral aneurysms, although none ruptured. BO-199 had mild right microphthalmia. All patients, except one (BO-215), had a positive family history of cystic kidney disease. Segregation analysis of the identified variants was performed in 6 out of 14 families (Figure 1).

**Figure 2:**
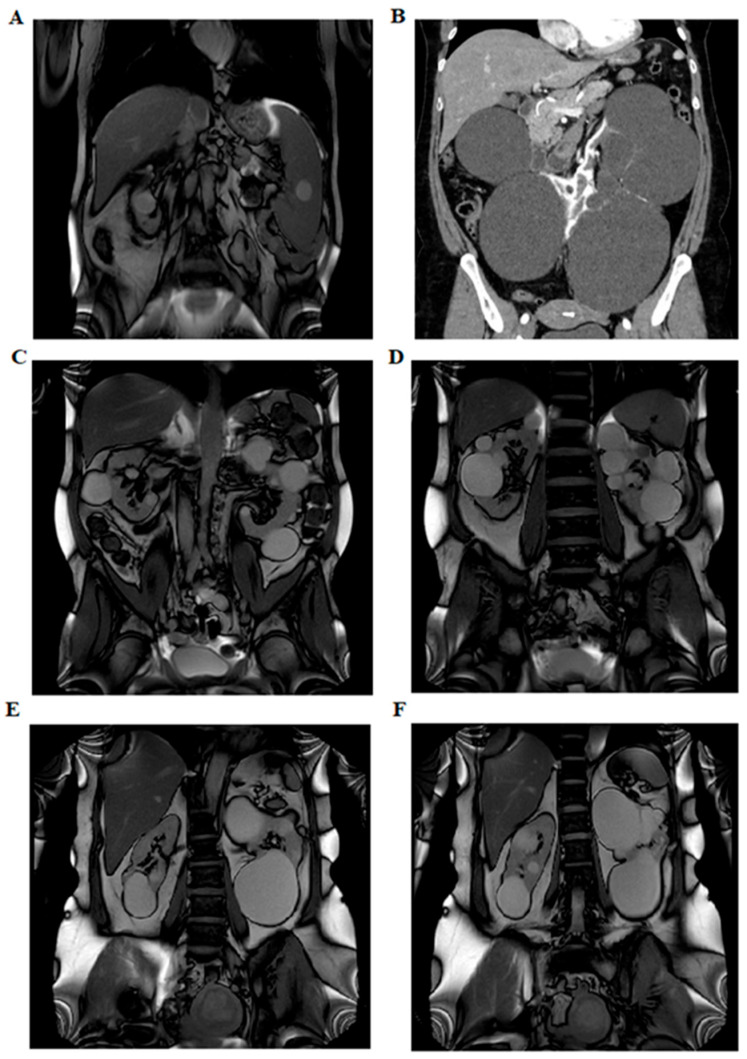
Abdominal imaging performed with nuclear magnetic resonance imaging and computed axial tomography. Panel (**A**) shows left ptosed kidney in left lumbar iliac spine with extra-rotated pelvis and multiple bilateral renal cysts (BO-183). Panel (**B**) shows several bilateral cortical renal cysts (B0-214). The panels (**C**,**D**) show multiple bilateral kidney cysts and exophytic cysts (BO-213). The panels (**E**,**F**) show few large renal cysts and no hepatic cysts (BO-216).

In five of these families, the respective variant co-segregated with the cystic phenotype. In family BO-217, the c.2766_2768 + 1del variant was absent in the affected mother, who instead carried an *ALG8* variant: c.980C > G (p.Thr327Arg), absent in the proband, classified as a VUS (CADD: Benign Moderate (21.7)). Abdominal MRI images of three patients (BO-183, BO-214, and BO-216) showing multiple bilateral cortical renal cysts are provided in Figure 2.

## 4. Discussion

ADPKD is the most common inherited cause of end-stage renal disease (ESRD) worldwide, with an estimated birth prevalence of approximately 1 in 1000. The two primary genes associated with ADPKD are *PKD1* and *PKD2*. However, additional genes, including *IFT140*, *GANAB*, *DNAJB11*, *ALG8*, and *ALG9*, have been identified in association with rarer ADPKD phenocopies. Variants in these newly recognized disease-associated genes should be considered in cases presenting with atypical imaging patterns (e.g., unilateral, asymmetric, segmental, lopsided, or bilateral cystic disease with unilateral or bilateral kidney atrophy) or in the absence of typical extrarenal manifestations, such as liver cysts [30]. The IFT140 gene is emerging as the third most common and frequently mutated gene in individuals with ADPKD, with a clinical picture that is usually at the milder end of the phenotypic spectrum: ESRD is rare but can occur in older individuals [10,11,12,17,30]. Due to the mild phenotype of polycystic kidney disease associated with the *IFT140* gene, renal disease in parents may go unnoticed. Consequently, patients lacking a documented family history are more likely to harbor pathogenic variants in the *IFT140* gene [31].

In this study, we report the results of a tNGS analysis of cystogenes responsible for classic and atypical ADPKD obtained in a cohort of 218 consecutive unrelated individuals who were tested in a single laboratory. As expected, *PKD1* and *PKD2* are the most prevalent genes, with a causative variant identified in 111 index-positive cases (83.5%), as shown in Table S2. The *IFT140* gene is confirmed to be the third most frequently mutated gene in these patients, with 14 unrelated patients out of 218 (6.5%). In addition, we provide a detailed clinical description of these 14 Italian families (Table 1), including the second pediatric IFT140-related phenotype, the first with a single nucleotide variant (SNV). At the time of this publication (March 2025), the only reported pediatric case in the literature was that of a 6-year-old girl with cystic nephroma and a heterozygous deletion of exon 13 in the *IFT140* gene. The molecular diagnosis in the patient was established after nine years, as the initial NGS sequencing had yielded negative results [32]. As can be seen from Seeman’s study, a limitation of our study is the lack of *IFT140* structural variants investigation in atypical negative cases. MLPA *IFT140* analysis could improve the detection rate of our diagnostic test.

Our results support the findings of previous studies: heterozygous pathogenetic *IFT140* variants are the third genetic cause of ADPKD, and liver involvement is rare (BO-215) and many carriers show atypical features in renal cyst size and locations, such as large, exophytic cysts (BO-183), with a total kidney volume not increased and preserved renal function compared to classical ADPKD caused by *PKD1/2* variants. Renal functional decline occurs only late in life, in contrast to what happens in patients with the classic ADPKD form. The prevalence of arterial hypertension is comparable to that observed in patients with pathogenic variants in *PKD1/2*. None of our patients had a cardiac phenotype (DCM) as described by Salhi S [11], but four showed mitral valve prolapse. Interestingly, 5/14 patients (35.7%) exhibited a hyperuricemic trait. The coexisting presence of CKD in three of them is a possible influencing factor.

To the best of our knowledge, this study reports the highest incidence of *IFT140* LOF variants in a cohort of subjects with cystic nephropathy. A recent large European cohort reported *IFT140* LOF variants in seventy-five individuals (from 61 independent families) among 2797 with ADPKD-like phenotypes [33]. The prevalence is less than 3%, similar to a distinct Italian cohort (2.3%) [12], but much lower than what we report here (6.4%). One possible explanation may be that our cohort is enriched with atypical cases, mirroring an enhanced interest of clinical nephrologists to identify the origin of renal cystic phenotypes and a frequent request for genetic tests.

Among the 11 variants identified in this case series, ten are loss of function, four of which are recurrent, and one missense. Five are novel variants, while six are reported in population databases (gnomADv4.1.0). In this context, as many as 7 of the 11 variants identified in our cohort are located between intron 19 and exon 23, suggesting that there may be a mutational hotspot.

The only missense variant, c.2797G > A (p.Glu933Lys), was classified as VUS and was present in proband BO-216 together with p.Arg307Ter. Unfortunately, the phase is unknown because no family members could be tested, but the phenotype was mild (few large kidney cysts at 67 years with normal renal function). Another individual, BO-199, had an *IFT140* LOF variant (p.Arg834Ter) and a missense variant in *PKD1* (p.Asn804Ser) classified as VUS. It is intriguing to note that both parents were known to have cystic nephropathy, but the father died at 67, and the mother, 80 years old, was not available for testing, but the presence of renal cysts was confirmed. This patient seemingly had a mild phenotype (bilateral kidney cysts, normal renal function at 57 years) but presented microhaematuria and was on regular follow-up. We also report a subject with a dual molecular diagnosis, BO-133, who had chronic kidney disease (stage IV at 78 years) with multiple bilateral cysts and diabetes mellitus: he had a frameshift variant in *IFT140*, but he was also found to have a whole gene deletion of *HNF4A* associated to MODY type 1. The association of MODY with a renal cystic phenotype caused by an *IFT140* LOF variant might have accelerated the decrease in renal function.

Segregation analysis could be performed in six families, and the respective variant was found to co-segregate with the cystic phenotype in five families. In one family (BO-217), the c.2766_2768 + 1del variant was not present in the affected mother who had a classic ADPKD phenotype (multiple cysts in kidney and liver, ESRD at 55 years), but no variants were identified in *PKD1* or *PKD2*; she was found to carry an *ALG8* variant (p.Thr327Arg), absent in the proband, but this SNV cannot be definitely associated to the phenotype at the moment (it is classified as VUS with a CADD score benign moderate [21.7]).

Overall, genetic factors are increasingly recognized to contribute significantly to cystic nephropathies, even in mild/atypical cases where variants in genes other than *PKD1* and *PKD2* are more frequently identified. Among these genes, *IFT140* is emerging as an important phenotypic driver. It is likely that several milder cases will turn out to be oligogenic with distinct genetic contributions. At the same time, in the interplay among different cystogenes, *IFT140* variants may well be considered candidates to modify the disease course of *PKD1*- and *PKD2*-related ADPKD.

## 5. Conclusions

This study confirms that *IFT140* LoF variants represent a strong contributor to renal cyst development. Kidney function is usually preserved even in older individuals, but a careful follow-up is warranted. The possible association with extrarenal manifestations is not common and needs larger clinical cohorts to be ascertained, but this study does not confirm the association with dilated cardiomyopathy. Hyperuricemia appears to be a new trait associated with this condition. Further studies in larger cohorts will be needed to confirm this evidence. Finally, expanding genetic testing beyond *PKD1* and *PKD2* is crucial in order to achieve an accurate diagnosis and should definitely include *IFT140*.

## Figures and Tables

**Table 1:** IFT140 variants description. LP: likely pathogenic; P: pathogenic; VUS: variant of unknown significance; FS: frameshift; N: nonsense; M: missense; S: splicing; NA: not applicable; PS: Pathogenic Strong; PSS: pathogenic supporting.

Position (hg19)	HGVS Coding (NM_014714.4)	HGVS Protein (NP_055529.2)	Exon/IVS	Varsome	Franklin	ClinVar	Impact	CADD	GnomAD (4.1.0)	ID
chr16:1657248	c.20_21insT	p.(Gln8ProfsTer82)	3	LP	LP	NA	FS	NA	Absent	BO-183
chr16:1637289	c.919C > T	p.(Arg307Ter)	9	P	P	P	N	PS (38)	2	BO-216
chr16:1630783	c.1501C > T	p.(Arg501Ter)	13	P	P	P	N	PS (39)	7	BO-215 BO-218
chr16:1616193	c.1863_1866del	p.(Glu623ArgfsTer20)	16	P	P	P	FS	NA	11	BO-214
chr16:1607935	c.2399 + 1G > T	p.?	ivs19	P	P	P	S	PS (35)	474	BO-049 BO-213
chr16:1576697	c.2500C > T	p.(Arg834Ter)	20	P	P	P	N	PS (53)	17	BO-199 BO-207
chr16:1576643	c.2545_2554del	p.(Val849TrpfsTer24)	20	LP	LP	NA	FS	NA	Absent	BO-071
chr16:1575974	c.2682delCinsAA	p.(His894GlnfsTer58)	21	P	P	P	FS	NA	Absent	BO-178
chr16:1575886	c.2766_2768 + 1del	p.?	21	P	P	LP	S	NA	Absent	BO-217
chr16:1575299	c.2797G > A	p.(Glu933Lys)	22	VUS	VUS	VUS	M	PSS (28.3)	23	BO-216
chr16:1574902	c.2880G > A	p.(Trp960Ter)	23	LP	LP	NA	N	PS (49)	Absent	BO-101 BO-126

**Table 2:** Clinical renal and extrarenal manifestations of heterozygous IFT140 patients. M: male; F: female; CKD: chronic kidney disease; MVP: mitral valve prolapse; MODY: Maturity Onset Diabetes of the Young.

ID	Sex	Family History of Kidney Disease	Age at Genetic Test	Age at Clinical Diagnosis	CKD Stage	Bilateral Renal Cysts	Liver Cysts	Heart	Hypertension	Urinary Findings	Uric Acid (Baseline)	Other Clinical Features
BO-071	M	yes	63	50	I	yes	none	none	yes	none	unknown	none
BO-126	M	yes	16	15	I	yes	none	none	no	none	unknown	none
BO-199	F	yes	57	40	I	yes	none	none	no	microhematuria	5.1 mg/dL	right microphthalmia, Bence-Jones proteinuria
BO-049	M	yes	39	34	I	yes	none	none	no	none	4.3 mg/dL	none
BO-217	F	yes	49	45	II	yes	none	MVP	no	none	4.4 mg/dL	none
BO-178	M	yes	50	47	II	yes	none	none	yes	microhematuria and albuminuria	8 mg/dL	glucose intolerance
BO-214	F	yes	54	41	II	yes	none	none	yes	none	4.3 mg/dL	two posterior cerebral aneurysms
BO-216	F	yes	67	63	II	yes	none	MVP	yes	none	3.6 mg/dL	intracranial aneurysm
BO-101	M	yes	59	30	III	yes	none	MVP	yes	none	6.4 mg/dL	gout
BO-207	M	yes	60	59	III	yes	none	unknown	no	none	7.5 mg/dL	none
BO-213	M	yes	64	64	III	yes	none	MVP	yes	proteinuria	7.4 mg/dL	none
BO-215	M	none	74	69	III	yes	yes	none	yes	none	5.2 mg/dL	none
BO-183	M	yes	78	78	IV	yes	none	unknown	yes	albuminuria	5.6 mg/dL	MODY, type 1
BO-218	F	yes	71	30	IV	yes	none	unknown	yes	none	13.3 mg/dL	none

## Data Availability

The data presented in this study are available on request from the corresponding author upon reasonable request.

## Supplementary Materials

The following supporting information can be downloaded at https://www.mdpi.com/article/10.3390/genes16050472/s1, Table S1: List of genes sequenced by tNGS; Table S2: Genetic results of tNGS in all patients.

## Appendix A

### Appendix A.1. Pedigree Clinical Features

The section below summarizes the clinical characteristics of the families described in our study.

#### Appendix A.1.1. Pedigree BO-049

The proband was a 39-year-old man who underwent abdominal US for recurrent fevers at the age of 34 and was found to have multiple bilateral kidney cysts but no liver cysts. He started medical therapy for arterial hypertension at 37. Renal function was normal at the last evaluation (creatinine 0.92 mg/dL, eGFR 104 mL/min/1.73 m^2^, uric acid 4.1 mg/dL), and urinalysis was normal. The right kidney measured 10.7 cm with multiple cysts and an exophytic cyst measuring 10 cm; the left kidney was totally subverted by cysts. His mother was 62 years old and had multiple bilateral kidney cysts, casually identified, with normal renal function. None of their family members developed ESRD. NGS sequencing identified a heterozygous canonical splicing variant (c.2399 + 1G > T) in the *IFT140* gene. Maternal DNA was not available, but the same variant was reported in family M132 in Senum et al. [10].

#### Appendix A.1.2. Pedigree BO-071

The proband, a 63-year-old man, had hypertension and bilateral kidney cysts found around the age of 50. At the last clinical evaluation, his renal function was preserved. The proband had one son and one daughter. His son showed a complex phenotype characterized by developmental delay, Ito’s hypomelanosis, and the onset of epileptic seizures from 17 years of age. He also had arterial hypertension and, in 2022, underwent an abdominal ultrasound, which showed bilateral kidney cysts. His daughter also had kidney cysts, while his mother’s proband, who died of a stroke at 87, had a clinical diagnosis of polycystic kidney. In the suspicion of autosomal dominant polycystic kidney disease, NGS sequencing identified a heterozygous frameshift c.2545_2554del (p.Val849TrpfsTer24) variant in the *IFT140* gene. Segregation analysis in his son was positive, while it has to be performed in his daughter.

#### Appendix A.1.3. Pedigree BO-101

A 59-year-old man had arterial hypertension from the age of 30 and suffered two acute gout attacks in 2021. The following year, after a hypertensive crisis, he performed an abdominal ultrasound, which showed the presence of multiple bilateral cysts altering the renal eco-structure. Blood tests showed a slight impairment of renal function and hyperuricemia. The patient reported that his brother had some renal cysts from the age of 55, and a paternal aunt, who died at 80 because of cardiovascular issues, had chronic kidney disease. The NGS panel highlighted the presence of a heterozygous IFT140 variant: c.2880G > A (p.Trp960Ter). No segregation analysis could be performed in this family.

#### Appendix A.1.4. Pedigree BO-126

The proband was a 15-year-old boy who had been followed up by the Pediatric Nephrology Unit of IRCCS Azienda Ospedaliero-Universitaria of Bologna for bilateral kidney cysts with normal renal function. The most recent abdominal MRI scan showed a 30 × 23 mm Bosniak type IIF medullary renal cyst with a septum within the right kidney and some bilateral cortical and parapelvic cysts. Renal function was normal (creatinine 0.74 mg/dL) at the last evaluation (16 years old). He is the firstborn of apparently healthy parents, except for the previous finding of a single kidney cyst in his father; his younger sister underwent an abdominal US scan, which proved normal. The NGS panel identified a nonsense heterozygous variant c.2880G > A (p.Trp960Ter) in the *IFT140* gene, classified as Likely Pathogenic. Segregation analysis showed that the variant was inherited by the father, who only had a single kidney cyst with no renal impairment. The paternal grandmother recently tested positive for *IFT140* segregation analysis. She has suffered from arterial hypertension since the age of 50 and has been taking allopurinol for the last two years. A recent abdominal ultrasound showed no hepatic lesions but some parapelvic cysts on the right kidney (2.2 cm) and a hyperechogenic left kidney with a polar cyst (2.7cm) and suspected cyst in the lower middle third (4.3 × 2.8cm).

#### Appendix A.1.5. Pedigree BO-178

The proband was a 50-year-old man with stage I–II chronic kidney disease with microalbuminuria and microhematuria. Abdominal ultrasounds showed few bilateral renal cysts, the largest on the right measuring 16 mm; the liver was increased in size, with regular profiles and thickened structure, but there was no evidence of focal lesions or cysts. He presented preserved renal function with creatinine 1 mg/dL, eGFR 83 mL/min/1.73 m^2^. He also presented glucose intolerance, hyperuricemia, arterial hypertension, hypercholesterolemia, and psoriatic spondylarthritis. The father, aged 80, had bilateral renal cysts since a young age, but the renal function is still preserved. He has a heart disease and has type 2 diabetes mellitus. His mother also has DM2. The NGS panel identified no pathogenic *PKD1* or *PKD2* variants in the proband, but a nonsense heterozygous variant c.2682delCinsAA (p.His894GlnfsTer58) in *IFT140* gene was detected. Segregation analysis showed the presence of the variant in the affected father and in the son currently unaffected.

#### Appendix A.1.6. Pedigree BO-183

The index case was a 78-year-old man followed by the Nephrology Unit of IRCCS Azienda Ospedaliero-Universitaria of Bologna for chronic kidney disease at the G4-A3 stage, ptotic left kidney, and multiple bilateral kidney cysts. In 2009, he performed percutaneous angioplasty positioning of a non-medicated STENT in the right coronary artery and a medicated STENT in the postero-lateral branch. In 2020, he was hospitalized again for acute myocardial infarction. He has diabetes mellitus. In 2023, an abdominal ultrasound showed two cysts of 28 mm and 26 mm at the upper third and at the bottom of the right kidney, respectively. The left kidney was ptotic with a thinned cortical portion, loss of cortical-medullar gradient, and two cysts (the largest one of 33 mm). Some cysts were also reported in the spleen (the largest was 21 mm). The proband had a sister who died at 80, who needed dialysis treatment, and a daughter in apparent good health.

NGS sequencing was performed and identified a heterozygous variant c.20_21insT (p.Gln8ProfsTer82) in the *IFT140* gene. At the same time, MLPA analysis of genes associated with diabetes mellitus and kidney/extra kidney anomalies showed a heterozygous deletion of *HNF4A*. The heterozygous *IFT140* variant and *HNF4A* deletion were both absent in his healthy daughter.

#### Appendix A.1.7. Pedigree BO-199

The proband was a 57-year-old woman with a history of bilateral renal cysts diagnosed at the age of 40 years. The cysts were primarily located in the upper pole of the right kidney, where they formed a cluster measuring 10.5 × 7.5 cm. She had normal renal function (creatinine 0.74 mg/dL, eGFR 91 mL/min/1.73 m^2^), while urine analysis showed microhematuria. An abdominal CT scan performed during nephrological follow-up ruled out the presence of cysts in other organs. The patient reported having undergone a brain MRI, which revealed mild right microphthalmia in the absence of other brain abnormalities. Her father had died at 67, likely due to heart disease, but he had renal cysts, and his mother also had renal cysts. The patient’s mother had Bosniak type II cysts in the left kidney and bilateral renal cortical cysts. Suspecting an autosomal dominant polycystic kidney disease, an NGS panel of related genes was performed and identified two different heterozygous variants. A nonsense mutation in *IFT140* gene: c.2500C > T (p.Arg834Ter) classified as pathogenic and a VUS in *PKD1*: c.2411A > G (p.Asn804Ser).

#### Appendix A.1.8. Pedigree BO-207

The proband was a 59-year-old man with an incidental finding of creatinine increase. The patient underwent abdominal ultrasonography, which revealed the presence of multiple large cystic formations with cortical exophytic development. He had an impaired kidney function (creatinine 1.86 mg/dL, eGFR 38 mL/min/1.73 m^2^). Upon collecting the family history, it was found that his mother had diabetes mellitus (age 87 years old), and his father, who died at age 58 from ischemic heart disease, had cystic nephropathy. He had six siblings, the eldest of whom reportedly manifested pancreatitis at about age 65 and later developed renal failure requiring dialysis treatment. The patient presented polyuria and hyperuricemia, currently compensated with drug treatment. NGS sequencing was performed and identified a heterozygous truncating variant c.2500C > T (p.Arg834Ter) in the *IFT140* gene. No segregation analysis could be performed in this family.

#### Appendix A.1.9. Pedigree BO-213

The proband was a 64-year-old man who first came to our clinic in May 2024 for impaired renal function and proteinuria. None of his family members presented with kidney disease. In his personal history, the patient presented hypertension, hypercholesterolemia, and Chronic Obstructive Pulmonary Disease (COPD). At the last assessment, the serum creatinine was 1.31 mg/dL, and the eGFR was 58 mL/min/1.73 m^2^. Proteinuria of 1.2 g/die was also reported. An ultrasound performed one year before showed the presence of an atypical form of polycystic kidneys, data that was confirmed by a subsequent MRI (Figure 2). The cardiologic assessment was carried out with a heart ultrasound, where mild mitral and aortic insufficiencies were reported. NGS sequencing identified the same heterozygous canonical splicing variant (c.2399 + 1G > T) in the *IFT140* gene, previously reported in BO-049.

#### Appendix A.1.10. Pedigree BO-214

The proband was a 47-year-old woman with a history of bilateral renal cysts diagnosed at the age of 41 years and a recent diagnosis of arterial hypertension in need of pharmacological treatment. She presented with several bilateral renal cysts, mostly cortical, with the two major ones measuring 14 cm and 16 cm in the right and left kidney, respectively. At the last assessment, the serum creatinine was 0.72 mg/dL and the eGFR was 91 mL/min/1.73 m^2^. She was also diagnosed with two posterior cerebral aneurysms. From the cardiological point of view, she underwent an echocardiography that proved to be normal. Upon reconstruction of family history, the proband had a sister with kidney cysts and a cerebral aneurysm and a first-degree cousin who underwent kidney transplantation at the age of 30, but there is no clinical documentation available for him. Suspecting an autosomal dominant polycystic kidney disease, an NGS panel of related genes was performed. The NGS panel identified a nonsense heterozygous variant c.1863_1866del (p.Glu623ArgfsTer20) in the *IFT140* gene. This variant is currently classified as Pathogenic. The affected sister shared the same variant.

#### Appendix A.1.11. Pedigree BO-215

The proband is a 74-year-old man who showed a phenotype characterized by the presence of liver and kidney cysts. In 2013, at 63 years old, he performed a CT scan, which revealed multiple cortical renal cysts that allowed a diagnosis of atypical polycystic kidney disease. An MRI performed in 2019 confirmed this data and also found the presence of cysts in the liver. The last values of creatinine and eGFR were 1.41 mg/dL and 49 mL/min/1.73 m^2^, respectively, showing impaired renal function (CKD stage IIIa). He also suffered from a myocardial infarction at 66. No other family members were reported to have kidney cysts and there was no familiarity with renal diseases. The mother died at 84 years old with an unspecified cardiomyopathy, while the father died at 79 years old after trauma. The sister died at 72 y due to bladder cancer. The proband has a healthy daughter. Despite the lack of familiarity, it was recommended to analyze *PKD1* and *PKD2* genes, but the sequence and MLPA analyses resulted in normal. Afterward, a gene panel was performed, detecting a heterozygous pathogenic variant in *IFT140* c.1501C > T (p.Arg501Ter).

#### Appendix A.1.12. Pedigree BO-216

The proband was a 67-year-old woman with few large renal cysts, and her liver was unaffected. Renal function at the last evaluation showed normal values, with a serum creatinine of 0.83 mg/dL (eGFR 74 mL/min/1.73 m^2^). She had arterial hypertension well controlled by treatment. She also presented an intracranial aneurysm. She underwent a cardiological assessment for Tako-Tsubo syndrome. The family history was positive for ADPKD: her brother, mother (deceased at age 90), and two maternal uncles were affected. None of her family members reached ESRD. Her brother also suffered from arterial hypertension and a myocardial infarction at 61 years of age. The NGS panel identified a pathogenic nonsense heterozygous variant c.919C > T (p.Arg307Ter) in the *IFT140* gene. The analysis also identified a missense heterozygous variant c.2797G > A (p.Glu933Lys) in the IFT140 gene, currently classified as VUS. Segregation analysis has not yet been performed.

#### Appendix A.1.13. Pedigree BO-217

The proband, a 45-year-old woman, was evaluated for suspected adult polycystic kidney disease: she had multiple cortical and subcortical cysts, with the largest in the right kidney measuring approximately 85 × 62 × 77 mm. At the last evaluation, the patient did not exhibit proteinuria or microhaematuria, and her renal function was normal (serum creatinine of 0.82 mg/dL and eGFR of 84 mL/min/1.73 m^2^). Echocardiography revealed mild mitral regurgitation, and brain MRI results were normal. The proband’s mother had kidney and liver cysts, started dialysis at age 55, and later received a kidney transplant. Additionally, an uncle (the mother’s brother) was reported to have hepatorenal disease and nephromegaly. Genetic testing for PKD1 and PKD2 was performed but did not reveal any pathogenic alterations. The NGS panel identified a truncating heterozygous variant c.2766_2768 + 1del in the IFT140 gene. The segregation analysis was performed on her affected mother, but was negative. The NGS analysis of her mother identified a variant in the ALG8 gene (p.Thr327Arg), classified as VUS, absent in our proband.

#### Appendix A.1.14. Pedigree BO-218

The proband was a 70-year-old woman with a history of atypical polycystic kidneys diagnosed with an ultrasound at 30 years old. She also presented arterial hypertension and polymyalgia rheumatica. At the last assessment, serum creatinine was 1.43 mg/dL and eGFR was 37 mL/min/1.73 m^2^. At 69 years old, she suffered from deep vein thrombosis and a subsequent pulmonary embolism, for which she was treated. When assessing her family history, no precedent records of renal disease were found among her relatives. Her brother died from a not-specified cardiological disease, while her daughter’s ultrasound resulted in negative for renal cysts, although she had a small angiomyolipoma. The proband underwent genetic testing for PKD1 and PKD2, but no pathogenic variants were identified. Afterward, the NGS panel identified a heterozygous pathogenic variant c.1501C > T (p.Arg501Ter) in the IFT140 gene. No segregation analysis was performed in this family.
