# Supplementary material for: Clinical Relevance of IFT140 Loss-of-Function Variants in Development of Renal Cysts
Source: Genes (Basel). 2025 Apr 22;16(5):472. doi: 10.3390/genes16050472 (PMC12111500; doi:10.3390/genes16050472)
Supplement: Supplementary file 1 [file genes-16-00472-s001.zip › TableS1.pdf]

Table 1

Gene	Location	RefSeq	MIM Gene	Phenotype	Inheritance	Phenotype MIM number
PKD1	16p13.3	NM_001009944.3	*601313	Polycystic kidney disease 1	AD	173900
PKD2	4q22.1	NM_000297.4	*173910	Polycystic kidney disease 2	AD	613095
GANAB	11q12.3	NM_198335.4	*104160	Polycystic kidney disease 3	AD	600666
PKHD1	6p12.3-p12.2	NM_138694.4	*606702	Polycystic kidney disease 4, with or without hepatic disease	AR	263200
DZIP1L	3q22.3	NM_173543.3	*617570	Polycystic kidney disease 5	AR	617610
DNAJB11	3q27.3	NM_016306.5	*611341	Polycystic kidney disease 6 with or without polycystic liver disease	AD	618061
ALG9	11q23.1	NM_001077690.1	*606941	Gillessen-Kaesbach-Nishimura syndrome	AR	263210
PRKCSH	19p13.2	NM_002743.3	*177060	Polycystic liver disease 1	AD	174050
SEC63	6q21	NM_007214.5	*608648	Polycystic liver disease 2	AD	617004
ALG8	11q14.1	NM_024079.5	*608103	Polycystic liver disease 3 with or without kidney cysts	AD	617874
LRP5	11q13.2	NM_002335.4	*603506	Polycystic liver disease 4 with or without kidney cysts	AD	617875
HNF1B	17q12	NM_000458.4	*189907	Renal cysts and diabetes syndrome	AD	137920
UMOD	16p12.3	NM_001008389.3	*191845	Tubulointerstitial kidney disease, autosomal dominant, 1	AD	162000
REN	1q32.1	NM_000537.4	*179820	Tubulointerstitial kidney disease, autosomal dominant, 4	AD	613092
SEC61A1	3q21.3	NM_013336.4	*609213	Tubulointerstitial kidney disease, autosomal dominant, 5	AD	617056
BICC1	10q21.1	NM_001080512.3	*614295	Renal dysplasia, cystic, susceptibility to	AD	601331
IFT140	16p13.3	NM_014714.4	*614620	Short-rib thoracic dysplasia 9 with or without polydactyly	AD	266920
